# Immunological Characteristics of Hyperreactive Malarial Splenomegaly Syndrome in Sudanese Patients

**DOI:** 10.1155/2013/961051

**Published:** 2013-03-05

**Authors:** Tayseer Alkadarou, Ahmed Musa, Abedelgader Alkadarou, Mohamed S. Mahfouz, Marita Troye-Blomberg, Ahmed M. Elhassan, Ibrahim M. Elhassan

**Affiliations:** ^1^Institute of Endemic Diseases, University of Khartoum, Sudan; ^2^Faculty of Medicine, University of Khartoum, Sudan; ^3^Faculty of Medicine, Jazan University, P.O. Box 114, Jazan, Saudi Arabia; ^4^Department of Immunology, The Wenner-Gren Institute, Stockholm University, Sweden

## Abstract

Hyperreactive Malarial Splenomegaly (HMS) is defined as a massive enlargement of the spleen resulting from abnormal immune responses after repeated exposure to the malaria parasites. This study was carried out in Khartoum, Sudan. Sudan is considered to be one of the countries where HMS is quite prevalent. The objective of the study was to determine the incidence of HMS in patients who reported to the Omdurman Tropical Diseases Hospital (OMTDH) in Sudan and to investigate the basic laboratory and immunological characteristics of this condition in these patients. A cross-sectional study was carried out in OMTDH, and all patients with enlarged spleens were included in the study. Thirty-one out of 335 (9.3%) patients were diagnosed as having the HMS condition using international criteria for HMS diagnosis. The mean serum immunoglobulin M (IgM) levels in HMS patient groups were 14.3 ± 5 g/L, and this was significantly higher compared with geographically matched controls (*P* < 0.001). Immunoglobulin G (IgG) C anticircumsporozoite (CSP) antibody levels were higher in the HMS patients although the difference was not statistically significant, when compared with a group of patients with mild malaria. In comparison with naïve European controls, both the HMS and the mild malaria groups had significantly higher antimalarial antibody levels *P* < 0.001 and *P* < 0.01, respectively. Plasma levels of interleukin 10 (IL10) and interferon gamma (IFN**γ**) were significantly increased in the HMS patients compared with the healthy control donors (*P* < 0.05 and *P* < 0.01) for IL10 and IFN**γ**, respectively. The findings of this study suggest that HMS is one of the significant causes of tropical splenomegaly in Sudan. HMS is associated with significant elevations of circulating IgM and antimalarial IgG antibodies as well as IL10 and IFN**γ**.

## 1. Introduction

Hyperreactive Malarial Splenomegaly (HMS) is characterized by massive enlargement of the spleen in the tropics. The condition is prevalent in certain malarious regions of the Old World, mainly in Africa [1–5]. 

HMS seems to be associated with a high mortality; however, the natural history of HMS is not well documented. A 5-year-mortality rate of 50% has been reported in Uganda and New Guinea [6]. A mortality rate of 85% has been documented in hospitalized patients with gross splenomegaly. Whether HMS is responsible for this high mortality is a fact that still needs to be established [6].

*Clinical Features*. Symptoms of splenomegaly consist primarily of left upper quadrant pain with or without signs of hypersplenism dominating the clinical presentation of HMS. Early in the syndrome, the pain may be episodic and exacerbated by physical activity, which over time progresses in intensity and becomes persistent and debilitating. Haemolytic episodes associated with acute febrile illness or pregnancy can precipitate the course of HMS and, on occasion, be associated with sudden death. Physical examination usually reveals massive splenomegaly of the spleen often extending across the midline to the right side of the abdomen or downward to the left iliac fossa. The spleen is generally firm and nontender with a prominent notch and regular contour. Hepatomegaly, most commonly of the left lobe, often coexists and tends to parallel the size of the spleen. The pathogenesis of HMS is still not fully understood, yet there seems to be agreement consensus on the hypothesis postulated earlier by Fakunle and Ziegler that HMS pathogenesis results from IgM overproduction due to B lymphocytes stimulation by malaria antigen/mitogen [7, 8] as being the basis for the development of HMS. 

 However, the occurrence of HMS in tribal and family clusters suggests host genetic factors involvement in the control of the IgM overproduction seen in these patients. Hyperreactive malaria splenomegaly is only found in malaria endemic areas [9]. Malaria in Sudan is endemic and characterized by seasonal transmission in most parts of the country [10]. Currently there is no available information on the prevalence and incidence of HMS in Sudan, apart from recently published data in the eastern part of the country which indicated that HMS is a major cause of splenomegaly in this part. In this areas HMS constitutes 11% of the total of splenomegaly cases [11]. This study aims to elucidate the prevalence and immunological characteristics of HMS in Sudanese patients.

The unusual immunological features of HMS, as described earlier, might contribute to our understanding of the mechanisms involved in pathogenicity and immunity to malaria. 

## 2. Methods

### 2.1. Study Area

This study was carried out in the Ommdurman Tropical Diseases Hospital (OTDH), Khartoum, Sudan, where patients from different regions of Sudan are referred to.

### 2.2. Study Design

A cross-sectional study was carried out from January 2004 to December 2006. All patients (330 patients) presented to the outpatient clinic with enlarge spleen were included in the study. 

Ethical approval of this study was obtained from the ethical committee at the Institute of Endemic Diseases, University of Khartoum. Informed consent was obtained from all adults and in case of children from their parents or guardians. Patients with severe diseases were excluded from this study.

The diagnosis of HMS was based mainly on the standard criteria for diagnosis of HMS [12] which included physical examination such as massive splenomegaly and exclusion of other common causes of huge spleen, that is, history of hepatitis or alcohol abuse, exposure to schistosomiasis or leishmaniasis, a family history of haemoglobinopathies or clinical evidence of fever, jaundice, lymphadenopathy, hepatomegaly, and portal hypertension. Finally, the form included history of malaria in terms of number of attacks during the year, last clinical attack, and treatment received that was compiled for each patient. Total and differential white cell counts were carried out for all patients to exclude leukemia. Urine and stool samples were examined to exclude schistosomiasis brucellosis using the Widal test kit (Hansard Diagnostic Limited, UK). 

Furthermore, the immunological parameters in established HMS were measured and compared with mild malaria patients and control groups.

Sera from 33 patients with parasitologically confirmed mild malaria from highly endemic areas in central Sudan were used as positive controls for immunological assays.

Patients were interviewed, and a full clinical history was obtained using specially designed forms which included the name, sex, age, tribe, and address.

All cases with HMS were treated with chloroquine 300 mg weekly, in accordance with local treatment protocol, and they were instructed to report to the study team at the hospital for followup once a month for 3 months.

### 2.3. Blood Sampling

Ten mL of peripheral blood was collected from each patient by venipuncture into EDTA vacutainers; blood samples were collected into plain sterile containers to separate serum within 12 h of collection. Plasma samples were stored frozen at –20°C. The packed red blood cells were transferred into sterile tubes and stored at −40°C for DNA extraction. 

### 2.4. Parasitological Examination

Thick and thin films were prepared from all study subjects, stained with Giemsa's and examined under microscope. Films were considered negative after examination of 300 oil fields without detection of malaria parasites.

Ten mL of peripheral blood was collected from each patient by venipuncture into EDTA vacutainers; blood samples were collected into plain sterile containers to separate serum within 12 h of collection. Plasma samples were stored frozen at –20°C. The packed red blood cells were transferred into sterile tubes and stored at −40°C for DNA extraction. 

### 2.5. Polymerase Chain Reaction (PCR)

PCR analysis was carried out on samples collected from all patients for detection of malaria parasites; the primers specific for the polymorphic regions block 2 of merozoite surface protein 1 (MSP1) and block 3 of (MSP2) were designed and described previously. The two genes were amplified using nested PCR. An initial amplification of the outer regions of the two genes was followed by a nested PCR with allelic family specific primer pairs [13].

### 2.6. Haemoglobin Level

Haemoglobin concentration was estimated by Drabkin's method [14].

### 2.7. Immunological Methods

Measurement of total human IgM: total (IgM) was measured using the MININEPHTM Human IgM Kit Binding Site Limited, UK. Serum samples from 31 healthy Sudanese donors were included as controls in the analysis.

### 2.8. Circumsporozoite (CSP) Peptide ELISA

A synthetic peptide derived from different regions of the circumsporozoite protein was used in this study. This antigen was synthesized according to protocol established by [15, 16]. The peptide was conjugated with bovine serum albumin (BSA). The ELISA tests were carried out using the standard method described elsewhere [17].

Cutoff was determined as the mean plus 2 standard deviations of the optical density (OD) values obtained with sera from 10 European donors with no history of malaria exposure. Thirty-three sera from parasitologically confirmed *P. falciparum* infected patients from a highly endemic malaria area were used as positive controls. 

Determination of the plasma levels of interleukin 10 (IL10) and gamma interferon (IFN*γ*): plasma levels of IFN*γ* and IL10 cytokines were measured in the plasma samples collected from HMS patients and healthy control donors by double sandwich ELISA using commercially available R & D system Elisa kits (Germany). 

### 2.9. Treatment

All patients with HMS were treated with chloroquine 300 mg weekly in accordance with the standard treatment protocol and were instructed to report to the study team at the health centers for followup once a month for 3 consecutive months.

### 2.10. Statistical Analysis

Data entries were performed using the Excel program. The PASW 18.02 software programme was used for data analysis. Frequency distributions were obtained, and descriptive statistics were calculated, including central tendencies, standard deviations, and 95% confidence intervals. For comparing the two groups (malaria patients versus control patients), Student's *t*-test was used to evaluate whether there was a significant difference in the two studied groups in their immunological parameters. All statistical tests were two-sided; a level of *P* < 0.05 was used to indicate statistical significance. 

## 3. Results

### 3.1. HMS Patients

The age of patients ranged from 10 to 70 years; mean age was (26.3 ± 14.3) years. During the study period, 31 out of 335 (9.3%) patients were diagnosed with HMS patients. The clinical, haematological, and immunological characteristics of the patients are shown in Table 1. The mean spleen size was 12.7 ± 4.65. The mean liver size was 2.0 ± 2.67. The mean Hb level was 10.3 ± 2.5. The WBC count was 3050 ± 1062 (Table 1).

The patients with enlarged spleens were followed up for 3 months. Out of 33 HMS cases, 21 (70%) of them completed the clinical follow-up period up to day 90. In 14 patients (66%), the spleens were impalpable at the end of the 3 months.

### 3.2. Parasitological Results

No malaria parasites were detected in any of the HMS patients group using microscopic examination. However, polymerase chain reaction analysis indicated that one HMS case was harbouring low grade falciparum parasitaemia. All mild malaria patients were positive for *P. falciparum*. 

### 3.3. Immunological Results

#### 3.3.1. Total IgM Concentration

The mean serum IgM levels were compared between HMS patients and 33 (age, sex, and geographically matched) healthy adult controls. The mean IgM level of the control was 0.9 ± 0.6 g/L. The mean IgM level in the HMS patients group was 14.3 ± 5 g/L, a finding which was significantly higher as compared to the healthy control (*P* < 0.001).

#### 3.3.2. Anti-CSP Antibodies

The mean IgG anti-CSP antibody levels were 0.92 ± 0.31, 0.76 ± 0.4, and 0.34 ± 0.14 in HMS, mild malaria and negative controls, respectively.

The levels of anti-CSP antibody was significantly higher (*P* < 0.001 and *P* < 0.05) in HMS cases and in mild malaria patients, respectively, compared with negative control. No significant difference in the IgG anti-CSP antibody levels was seen between HMS and the malaria patient groups (*P* > 0.05).

#### 3.3.3. The Plasma Levels of IFN*γ* and IL10 in the Study Subjects

The plasma levels of IL10 and IFN*γ* are summarized in Table 2. The mean plasma levels of both IL-10 and IFN*γ* were significantly higher in the HMS patients, compared with the healthy control donors, *P* < 0.05 and *P* < 0.01, respectively.

## 4. Discussion

In the present study, 31 out of 335 splenomegaly patients (9.3%) presented with HMS conditions. This indicates that HMS is a significant cause of tropical splenomegaly in Sudan. 

In this study, the IgG anti-CSP antibody levels were found to be slightly higher (although not statistically significant) in the HMS patients compared with mild malaria patients (*P* > 0.05), whereas antimalarial IgG antibody levels were significantly increased in both HMS and mild malaria patients compared with malaria negative European controls (*P* < 0.01). This is in agreement with other previous studies carried out in areas characterized by stable malaria transmission [2, 18–21]. The results obtained in the current study confirm that high levels of anti-CSP antibodies are one of the immunological characteristics seen in HMS in stable as well as unstable malaria transmission areas. This feature might be suggestive of the role of anti-CSP antibody in protection against severe malaria in HMS patients. The production of IFN*γ* and IL10 cytokines in response to malaria antigens has been shown to be important in induction and maintenance of immunity to malaria in naturally exposed population. IFN-*γ* plays an important role in host defense against many infectious diseases [22]. Several studies have suggested that IFN-*γ* plays an important role in the regulation of immune responses during the course of infection by the activation of macrophages involved in both the intracellular and extracellular destruction of the parasite. A number of studies have shown that IFN-*γ* is associated with the pathogenesis, as seen in malaria-infected mice, and that the pathogenic effect of IFN-*γ* is counterbalanced by the anti-inflammatory cytokine, IL10 [23]; IL-10 is a pleiotropic immunomodulatory cytokine regulating not only Th1 but also Th2-type reactions in many instances [24, 25]. High levels of IL-10 observed during malaria episodes may be beneficial in reducing the inflammatory response, but it may also be detrimental by decreasing antiparasitic cellular immune responses [24]. We found that the plasma levels of IFN*γ* and IL10 cytokines were significantly elevated in HMS patients. This is the first paper of elevation of IFN*γ* and IL10 in HMS patients, suggesting that these cytokines might have an important role in the protection and/or pathogenesis of HMS conditions. 

## 5. Conclusion

This study shows that HMS is prevalent in Khartoum area of Sudan and that it constitutes a significant proportion of patients with splenomegaly. HMS patients in areas characterized by seasonal malaria develop high levels of anti-Csp antibody levels, and HMS is associated with an elevation of IFN*γ* and IL10 cytokines. Although these cytokines appear to play a role in the pathogenesis and protection in HMS, objective evidence regarding the exact phenomenon still needs to be determined.

## Figures and Tables

**Table 1:** Clinical hematological and immunological characteristic of HMS patients.

Male-female ratio	3 : 2
Mean of age ± SD (range) (years)	26.3 ± 14.3 (10–70)
Mean spleen size ± SD (cm)	12.7 ± 4.65
Mean liver size ± SD (cm)	2.0 ± 2.67
Mean haemoglobin concentrations ± SD (g/dL)	10.3 ± 2.52
Mean of white blood cells ± SD (range) (L)	3050 ± 1062
Mean of serum concentration of total IgM ± SD (g/L)	14.3 ± 5
Mean level of anti-CSP antibody levels (OD)	0.92 ± 0.31

**Table 2:** The plasma level of IFN*γ* and IL-10 concentrations (pg/mL) in healthy controls and HMS patients (confidence interval of mean level 95%).

	Mean	SD	95% CI for the mean	Significance
IFN*γ*				
Control	60	8.5	57.0–63.0	*P* < 0.05
HMS	75	14.2	70.0–80.0	
IL10				
Control	28	19.8	21.0–35.0	*P* < 0.01
HMS	42	25.5	35.0–53.0	
